# GP96 Drives Exacerbation of Secondary Bacterial Pneumonia following Influenza A Virus Infection

**DOI:** 10.1128/mBio.03269-20

**Published:** 2021-06-01

**Authors:** Tomoko Sumitomo, Masanobu Nakata, Satoshi Nagase, Yuki Takahara, Mariko Honda-Ogawa, Yasushi Mori, Yukako Akamatsu, Masaya Yamaguchi, Shigefumi Okamoto, Shigetada Kawabata

**Affiliations:** a Department of Oral and Molecular Microbiology, Osaka University Graduate School of Dentistry, Osaka, Japan; b Department of Oral Microbiology, Kagoshima University Graduate School of Medical and Dental Sciences, Kagoshima, Japan; c Department of Clinical Laboratory Science, Faculty of Health Sciences, Institute of Medical Pharmaceutical and Health Sciences, Kanazawa University, Kanazawa, Japan; d Advanced Health Care Science Research Unit, Innovative Integrated Bio-Research Core, Kanazawa University Institute for Frontier Science Initiative, Kanazawa, Japan; University of Illinois at Chicago

**Keywords:** *Streptococcus pneumoniae*, influenza virus, superinfection, pneumonia

## Abstract

Influenza A virus (IAV) infection predisposes the host to secondary bacterial pneumonia, known as a major cause of morbidity and mortality during influenza virus epidemics. Analysis of interactions between IAV-infected human epithelial cells and Streptococcus pneumoniae revealed that infected cells ectopically exhibited the endoplasmic reticulum chaperone glycoprotein 96 (GP96) on the surface. Importantly, efficient pneumococcal adherence to epithelial cells was imparted by interactions with extracellular GP96 and integrin α_V_, with the surface expression mediated by GP96 chaperone activity. Furthermore, abrogation of adherence was gained by chemical inhibition or genetic knockout of GP96 as well as addition of RGD peptide, an inhibitor of integrin-ligand interactions. Direct binding of extracellular GP96 and pneumococci was shown to be mediated by pneumococcal oligopeptide permease components. Additionally, IAV infection induced activation of calpains and Snail1, which are responsible for degradation and transcriptional repression of junctional proteins in the host, respectively, indicating increased bacterial translocation across the epithelial barrier. Notably, treatment of IAV-infected mice with the GP96 inhibitor enhanced pneumococcal clearance from lung tissues and ameliorated lung pathology. Taken together, the present findings indicate a viral-bacterial synergy in relation to disease progression and suggest a paradigm for developing novel therapeutic strategies tailored to inhibit pneumococcal colonization in an IAV-infected respiratory tract.

## INTRODUCTION

Influenza is an infectious respiratory disease, involved in more than 500,000 deaths worldwide each year. Although the severity of illness depends on various viral and host factors, influenza A virus (IAV), in particular, has an ability to adapt to the host, while reassortment events ensure constant generation of new virulent strains with unpredictable degrees of pathogenicity and transmissibility (1–3). In addition to primary influenza pneumonia, a secondary bacterial infection following influenza virus infection is also associated with high rates of morbidity and mortality in elderly as well as chronically ill individuals. During the 1918 influenza pandemic, Streptococcus pneumoniae was identified as the predominant pathogen in more than 95% of all fatal cases (4). Despite development of effective vaccines and potent antibacterial agents, during the 2009 H1N1 pandemic, bacterial pneumonia was a complication in one-quarter to one-half of severe and fatal cases (5). The underlying mechanism of the viral-bacterial synergy leading to disease progression has remained elusive, thus hampering production of effective prophylactic and therapeutic intervention options.

Nasopharyngeal pneumococcal colonization is a major predisposing factor related to viral upper respiratory tract infections such as influenza (6, 7). A preceding influenza virus infection can induce excess pneumococcal acquisition and carriage in the nasopharynx, which in turn promotes bacterial dissemination to the lungs. Although respiratory epithelium provides a physical barrier against most human pathogens, the influenza virus can replicate in epithelial cells, leading to direct damage of airway epithelium (8, 9). Virus-induced epithelial damage and exfoliation provide increased receptor availability for bacteria, resulting in establishment of bacterial colonization and onset of invasive diseases. For example, the influenza virus neuraminidase cleaves sialic acid glycoconjugates on airway epithelial cells as well as mucins, which facilitates not only bacterial adherence to cryptic receptors but also their proliferation in the upper respiratory tract (10). Among bacterial receptors appearing on cell surfaces during influenza virus infection, platelet-activating factor receptor (PAFR) gained attention as a possible therapeutic target (11). Extracellular PAFR binds to phosphorylcholine embedded in the cell walls of numerous respiratory bacterial pathogens, which subsequently accelerates lung bacterial burden and bacteremia and increases mortality risk. However, previous studies have found that genetic knockout or pharmacological inhibition of PAFR had no effect on the susceptibility of mice to secondary bacterial pneumonia, implicating multifaceted mechanisms related to the synergism between influenza viruses and bacterial pathogens (12, 13).

A dual viral-bacterial infection causes dysfunction of the epithelial-endothelial barrier and, consequently, exudation of fluids, erythrocytes, and leukocytes into alveolar spaces, leading to gas exchange impairment and severe respiratory insufficiency. Indeed, pulmonary edema and hemorrhage are commonly found in autopsy examinations (4). The physical barrier function of airway epithelium is provided by four types of cell-cell junctions: tight, adherens, and gap junctions, and desmosomes. Influenza virus-induced disruption of the pulmonary barrier is associated with a loss of integrity of claudin-4, a tight junctional protein (14). Moreover, interaction between the PDZ-binding motif of the avian influenza virus NS1 protein and the PDZ domain present in tight junctional proteins has been shown to destabilize epithelial junctional integrity (15). Although it is generally accepted that viral-induced epithelial cell damage allows for bacterial invasiveness, the molecular mechanisms involved in dysfunction of the alveolar epithelial barrier followed by bacterial dissemination remain largely unknown.

In the present study, novel findings showing that glycoprotein 96 (GP96), a host chaperone protein, is involved in the exacerbation of bacterial pneumonia following influenza A virus (IAV) infection are presented. Interactions of pneumococci with extracellular GP96 and integrins, whose surface expressions are mediated by the chaperone activity of GP96, were found to promote pneumococcal adherence to IAV-infected epithelial cells. Also, inhibition of GP96 rendered IAV-infected cells as well as tested mice less susceptible to S. pneumoniae infection. Accordingly, GP96 is considered to be a potential target for therapeutic strategies for treating patients with superinfection. Furthermore, to the best of our knowledge, the present results are the first to demonstrate that viral infection induces calpain/Snail1-dependent dysfunction of the pulmonary epithelial barrier, thus providing a route for secondary bacterial translocation into deeper tissues via paracellular junctions. Together, these findings suggest an underlying mechanism responsible for polymicrobial synergy in cases of secondary bacterial pneumonia.

## RESULTS

### Influenza A virus infection induces surface display of GP96 on alveolar epithelial cells.

**FIG 1:**
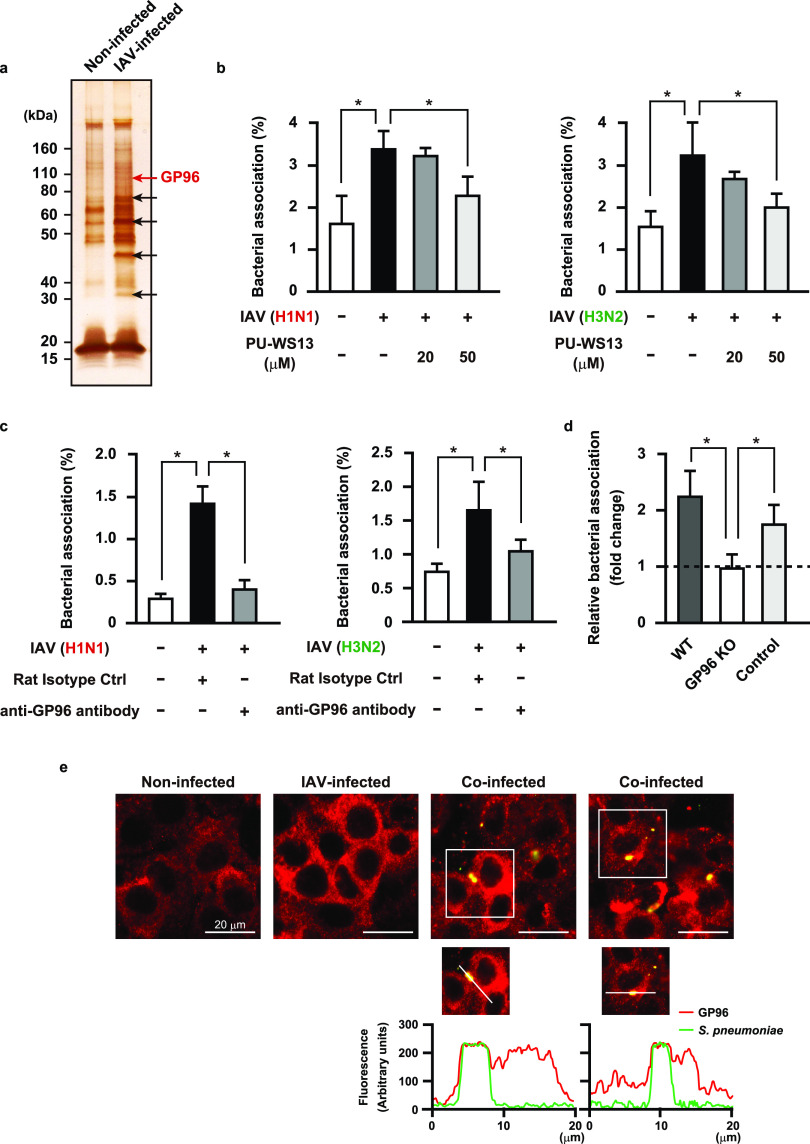
IAV infection-induced surface display of GP96 promotes pneumococcal adherence. (a) A549 cells were infected with 10^6^ PFU of IAV for 36 h and then treated with a membrane-impermeable biotinylation regent. Cell surface proteins were obtained using streptavidin beads and then subjected to SDS-PAGE and silver staining. Arrows indicate bands of upregulated surface proteins after IAV infection. Data shown are representative of at least three independent experiments. (b and c) A549 cells were infected with IAV (H1N1 or H3N2) for 1 h. Following washing steps, they were incubated for 36 h in the presence of PU-WS13 (b) or an antibody against GP96 (c). Next, IAV-infected cells were coinfected with S. pneumoniae D39 strain at a multiplicity of infection (MOI) of 5. At 2 h after infection, cells were lysed and cell-associated bacteria were recovered. Bacterial adherence rate was calculated as percentage of the inoculum. All experiments were performed in sextuplets with three technical repeats. Values are shown as the means ± standard deviations (SDs) from six wells from a representative experiment. (d) Effect of GP96 knockout (KO) on pneumococcal adherence. Bacterial association with IAV-infected cells was normalized to that with noninfected cells. For control cells with no mutation of the GP96 gene, cells cultured in the same manner as the GP96 knockout cells during selection were utilized. All experiments were performed in sextuplets with three technical repeats. Data are shown as relative bacterial association normalized to that with noninfected cells. Values are shown the means ± SDs from six wells from a representative experiment. *, *P* < 0.01 (b to d). (e) A549 cells were infected with IAV followed by S. pneumoniae infection. GP96 was labeled with anti-GP96 and Alexa Fluor 594-conjugated antibodies (shown as red in images), while S. pneumoniae was labeled with anti-serotype 2 capsule and Alexa Fluor 488-conjugated antibodies (shown as green in images). Images were analyzed using a confocal laser scanning microscope. Boxed area is magnified and shown in the lower panels. Graphs below the magnified images show fluorescence intensity profiles from a line crossing through the image of a coinfected cell. Coincidence of fluorescence intensity peaks, both red and green, indicates colocalization of S. pneumoniae with GP96. Data shown are representative of at least three separate experiments.

Host inflammatory response to a viral infection leads to increased or ectopic expressions of multiple proteins that serve as host receptors for bacteria. As a first step toward understanding the pathogenesis of bacterial pneumonia following influenza, we attempted to determine which proteins were exposed on the surfaces of alveolar epithelial cells following viral infection. Human A549 alveolar epithelial cells were infected with IAV A/FM/1/47 (H1N1) followed by exposure to a membrane-impermeable biotinylation reagent. Cell surface proteins were then obtained using streptavidin beads and subjected to SDS-PAGE and silver staining, which showed several different upregulated proteins on the surfaces of IAV-infected epithelial cells (Fig. 1a, arrows). Mass spectrometry analysis of these proteins revealed peptides corresponding to an endoplasmic reticulum protein, components of intermediate filaments, a glycolytic protein, and an oxidative stress-related protein. Among the host molecules, we focused on the human endoplasmic reticulum (ER) chaperone GP96, also referred to as GRP94 or endoplasmin, in further examinations. Although GP96 has been found to be mainly localized in the ER, abundant evidence presented indicates that it is also exposed on the surfaces of different cell types under particular conditions, such as infection, cell activation, and necrotic cell death (16). Surface-displayed GP96 is frequently exploited as a receptor for bacterial pathogens, including Listeria monocytogenes (17) and Neisseria gonorrhoeae (18). To examine whether exposed GP96 serves as a receptor for S. pneumoniae, bacterial adherence to the apical surfaces of epithelial cells infected with or without IAV was examined. S. pneumoniae showed more efficient adherence to IAV-infected than to noninfected cells, while the enhanced bacterial association was reduced by pretreatment with either a pharmacological inhibitor of GP96 (Fig. 1b, left) or anti-GP96 antibody (Fig. 1c, left). Pneumococcal adherence to noninfected cells was not affected by the addition of the inhibitor or antibodies (data not shown). Inhibition under both conditions also reduced pneumococcal adherence to epithelial cells following IAV A/Panama/2007/99 (H3N2) infection (Fig. 1b and c, right), indicating that the phenotype is not restricted to the IAV H1N1 subtype. On the other hand, a preceding IAV infection had no effect on the ability of S. pneumoniae to adhere to GP96 knockout cells (Fig. 1d), indicating it to be a critical factor for bacterial colonization on IAV-infected alveolar epithelial cells. To visualize GP96 distribution and pneumococci, nonpermeabilized cells were stained with anti-GP96 and anti-pneumococcal capsule antibodies, respectively (Fig. 1e). GP96 was poorly visualized on the surface of noninfected cells, whereas its surface expression was markedly increased in response to IAV infection. Notably, colocalization of pneumococci with GP96 was observed in superinfected cells. Based on these results, we speculated that redistribution of GP96 on epithelial surfaces caused by IAV infection has a crucial role in bacterial colonization in the lungs.

### Pneumococcal surface proteins AliA and AliB are determinants of bacterial adherence via the GP96 receptor.

**FIG 2:**
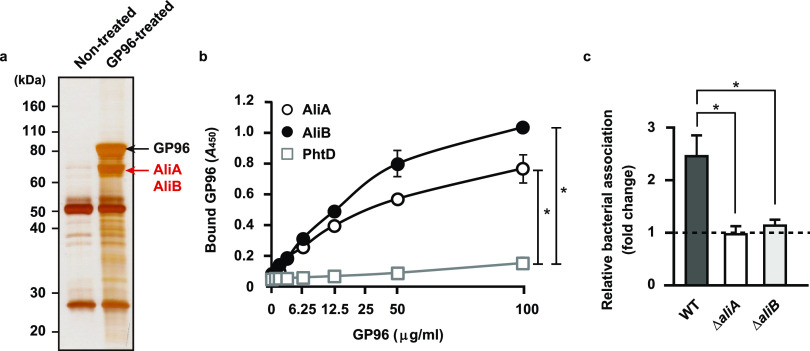
S. pneumoniae adheres to alveolar epithelial cells through interaction of pneumococcal surface proteins with GP96. (a) Proteins bound to GP96 were immunoprecipitated from pneumococcal cell wall fractions and then subjected to SDS-PAGE and silver staining. Data shown are representative of at least three separate experiments. (b) AliA, AliB, and PhtD, bacterial surface proteins, were immobilized on microtiter plates and then increasing amounts of GP96 were added. Bound GP96 was detected using an anti-GP96 antibody. All experiments were performed in sextuplets with three technical repeats. Values are shown as the means ± SDs from six wells from a representative experiment. *, *P* < 0.01. (c) Effects of deletion of *aliA* and *aliB* on pneumococcal adherence. Bacterial association with IAV-infected cells was normalized to that with noninfected cells. All experiments were performed in sextuplets with three technical repeats. Values are shown as the means ± SDs from six wells from a representative experiment. *, *P* < 0.01.

In the early stage of infection, bacterial pathogens secrete a variety of virulence factors that interact with host receptors for establishment of colonization. To identify bacterial factors responsible for GP96-mediated adherence to IAV-infected epithelial cells, pneumococcal cell wall fractions were obtained and reacted with recombinant GP96 protein, and then GP96-binding proteins were recovered by immunoprecipitation with an antibody against GP96. As shown in Fig. 2a, protein bands with an apparent molecular mass of approximately 70 kDa were identified as AliA (pI 5.00) and AliB (pI 5.24) by mass spectrometry analysis. AliA and AliB are components of the Ami-AliA/AliB oligopeptide permease complex and have functions related to bacterial colonization in the pharynx and lung (19). To examine the interactions of each with GP96, immobilized recombinant Ali proteins and the predominant pneumococcal surface protein PhtD (pI 5.22), used as a control, were incubated with serially diluted GP96 followed by detection with an anti-GP96 antibody. Even though the pI values of the tested proteins were nearly identical, GP96 was found to bind to the AliA and AliB proteins but not to PhtD (Fig. 2b). Furthermore, the binding affinity of Ali proteins to GP96 was evaluated using surface plasmon resonance (SPR) measurements. Equilibrium dissociation constants for the binding of AliA and AliB to GP96 protein were calculated by applying association and dissociation curves to a 1:1 Langmuir binding model (Table 1). SPR analysis revealed that both AliA and AliB bound to GP96 with a high affinity, with equilibrium dissociation constant (*K_D_*) values of 3.40 × 10^−8^ and 4.85 × 10^−8^ M, respectively. We next examined whether these pneumococcal Ali proteins function as adhesins for bacterial adherence to IAV-infected epithelial cells. Following IAV infection, the association of a wild-type (WT) strain to alveolar epithelial cells was increased by approximately 2.5-fold, whereas the adhesion activity of the *aliA* and *aliB* knockout mutants remained unchanged (Fig. 2c). These results suggest that S. pneumoniae utilizes AliA and AliB proteins as adhesins to interact with surface-displayed GP96 on IAV-infected cells, resulting in establishment of a secondary pneumococcal infection.

**TABLE 1:** Kinetic binding parameters for pneumococcal surface protein to GP96^*a*^

Ligand	*k*_a_ (M^−1^ s^−1^)	*K_d_* (s^−1^)	*K_D_* (M)
AliA	2.31 × 10^4^	7.85 × 10^−4^	3.40 × 10^−8^
AliB	2.09 × 10^8^	10.12	4.85 × 10^−8^

a *k_a_*, absorption rate constant; *K_d_*, dissociation constant; *K_D_*, equilibrium dissociation constant.

### Influenza virus infection-induced chaperone activity of GP96 promotes pneumococcal adherence to alveolar epithelial cells.

**FIG 3:**
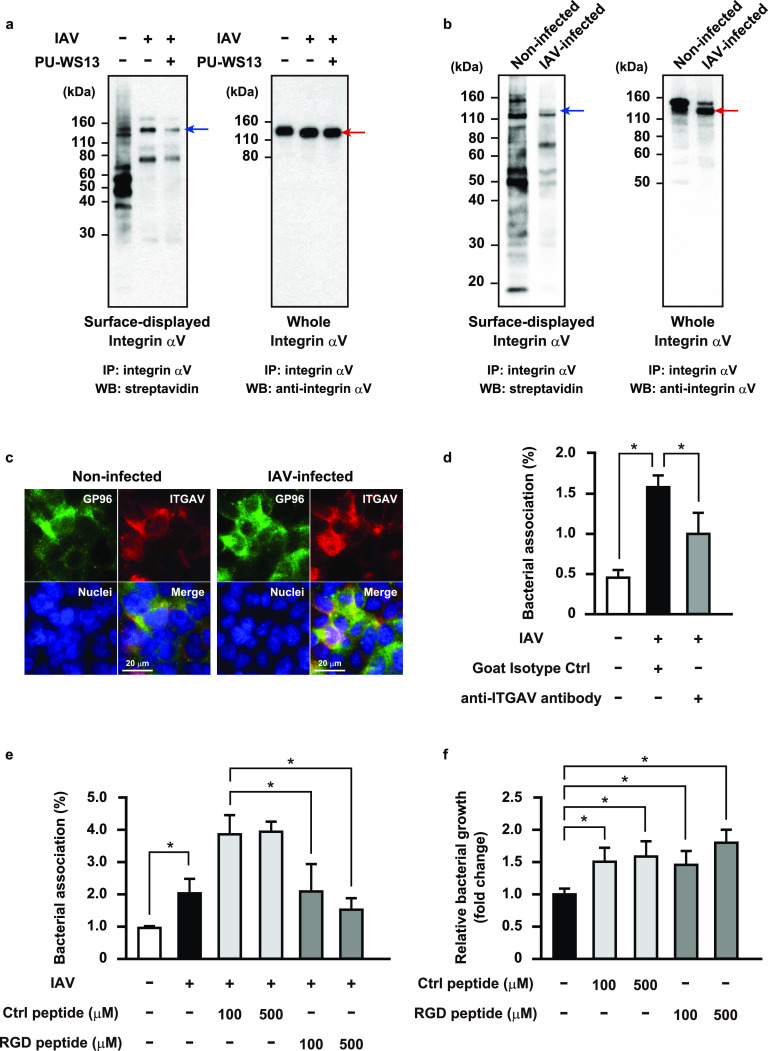
GP96-dependent surface display of integrin α_V_ associated with enhanced pneumococcal adherence following IAV infection. A549 cells (a) or GP96 knockout cells (b) were infected with IAV for 36 h in the presence or absence of PU-WS13 and then treated with a membrane-impermeable biotinylation reagent. Immunoprecipitation of cell lysates containing biotinylated surface proteins was performed using an antibody against integrin α_V_. Surface-displayed and whole-cell integrin α_V_ was detected using streptavidin and an antibody against integrin α_V_, respectively. Red arrows indicate integrin α_V_ bands. Blue arrows indicate predicted molecular weight of surface-displayed integrin α_V_. IP, immunoprecipitation; WB, western blotting. Data shown are representative of at least three separate experiments. (c) A549 cells were infected with IAV for 1 h. After transferring to fresh medium, incubation was continued for 36 h. GP96 was labeled with anti-GP96 and Alexa Fluor 488-conjugated antibodies, while integrin α_V_ was labeled with anti-integrin α_V_ and Alexa Fluor 594-conjugated antibodies. DAPI was used to stain DNA in the nucleus. Data shown are representative of at least three independent experiments. (d and e) A549 cells were infected with IAV for 36 h. Following washing steps, they were incubated with an antibody against integrin α_V_ (d) or RGD peptide (e) for 1 h, and then IAV-infected cells were coinfected with an S. pneumoniae strain at an MOI of 5. At 2 h after initiating infection, cells were lysed and cell-associated bacteria were recovered. Bacterial adherence rate was calculated as percentage of inoculum. All experiments were performed in sextuplets with three technical repeats. Values are shown as the means ± SDs from six wells from a representative experiment. (f) S. pneumoniae organisms were grown in cell culture medium containing the control or RGD peptide for 2 h, and then viable bacteria were recovered. Bacterial growth rate was calculated as percentage of inoculum. All experiments were performed in sextuplets with three technical repeats. Values are shown as the means ± SDs from six wells from a representative experiment. *, *P* < 0.01.

Notably, enhanced pneumococcal adherence by IAV infection was nearly completely abrogated in GP96 knockout cells but only partially in those cells treated with the anti-GP96 antibody (Fig. 1b and c), implying that additional host factors are involved in the enhanced bacterial association following IAV infection. GP96 is a molecular chaperone that has a key role in folding as well as surface expression of various integrin subunits and Toll-like receptors (TLRs) (20). Integrins are type I transmembrane heterodimeric proteins that mediate cell-cell and cell-extracellular matrix interactions. The major integrin ligand fibronectin (Fn) possesses a tripeptide arginine-glycine-aspartic acid (RGD) sequence that serves as the integrin recognition site. Bacterial pathogens responsible for secondary bacterial pneumonia, including S. pneumoniae, Streptococcus pyogenes, Staphylococcus aureus, and Haemophilus influenzae, utilize interactions with Fn integrins to associate with host cells (21–24). Thus, we speculated that IAV infection accelerates a GP96-dependent surface display of integrins that may augment bacterial association with epithelial cells. To examine in more detail, immunoprecipitation of cell lysates containing biotinylated surface proteins extracted from noninfected and IAV-infected epithelial cells was conducted using an antibody against integrin α_V_, a major α subunit involved in the pathogenesis of respiratory diseases. The expression levels of total integrin α_V_ were similar among all tested conditions, whereas a slight shift of the protein band potentially reflecting modification of a sugar moiety was observed in IAV-infected cells (Fig. 3a, right). A marked increase in surface-exposed integrin α_V_ was seen following IAV infection, which was largely hampered by introduction of the GP96 inhibitor (Fig. 3a, left). Next, we utilized GP96 knockout cells to further investigate the role of GP96 on the surface display of integrin αV. Integrin αV expression was detected in whole-cell fractions obtained from both noninfected and IAV-infected cells (Fig. 3b, right), whereas no protein band corresponding to integrin αV was observed in the cell surface protein fractions (Fig. 3b, left). Accordingly, it is considered likely that integrin α_V_ is exported to the surface of IAV-infected cells in a GP96-dependent manner. Immunofluorescence staining experiments also revealed that colocalization of integrin α_V_ with GP96 on the cell surface was more pronounced with IAV infection (Fig. 3c).

Next, we evaluated whether surface-exposed integrin α_V_ functions as a receptor for bacterial adherence to IAV-infected cells. As shown in Fig. 3d, S. pneumoniae demonstrated a greater level of adherence to IAV-infected cells than to noninfected cells, though that enhanced bacterial association was partially reduced by treatment with an anti-integrin α_V_ antibody. Anti-integrin α_V_ and goat isotype control antibodies had no effects on pneumococcal adherence to noninfected cells (data not shown). Furthermore, compared with the control peptide, an RGD-containing peptide significantly suppressed pneumococcal adhesion in a dose-dependent manner (Fig. 3e). In that inhibition assay, addition of the control peptide unexpectedly increased bacterial association with IAV-infected cells; thus, we examined whether pneumococcal growth was promoted in the presence of the control or RGD peptide. S. pneumoniae cells were cultured in cell culture medium (Dulbecco’s modified Eagle medium [DMEM] supplemented with 10% fetal bovine serum [FBS]) with or without the peptides, and viable pneumococcal counts were found to be significantly increased in the presence of either peptide (Fig. 3f). Thus, the increased bacterial association with IAV-infected cells was attributable, at least in part, to the peptide-driven bacterial growth. S. pneumoniae has evolved to gain ABC transporters used to acquire substrates for growth, such as micronutrients, amino acids, and peptides. Indeed, oligopeptide-binding proteins of the Ami-AliA/AliB permease have been shown to be responsible for the uptake of various oligopeptides, particularly those containing leucine and arginine (25, 26). In accordance with these results, the Fn integrin interaction was also found to be partly associated with pneumococcal adherence to IAV-infected alveolar epithelial cells.

### Influenza virus infection-induced Snail1 expression contributes to disruption of alveolar epithelial barrier.

**FIG 4:**
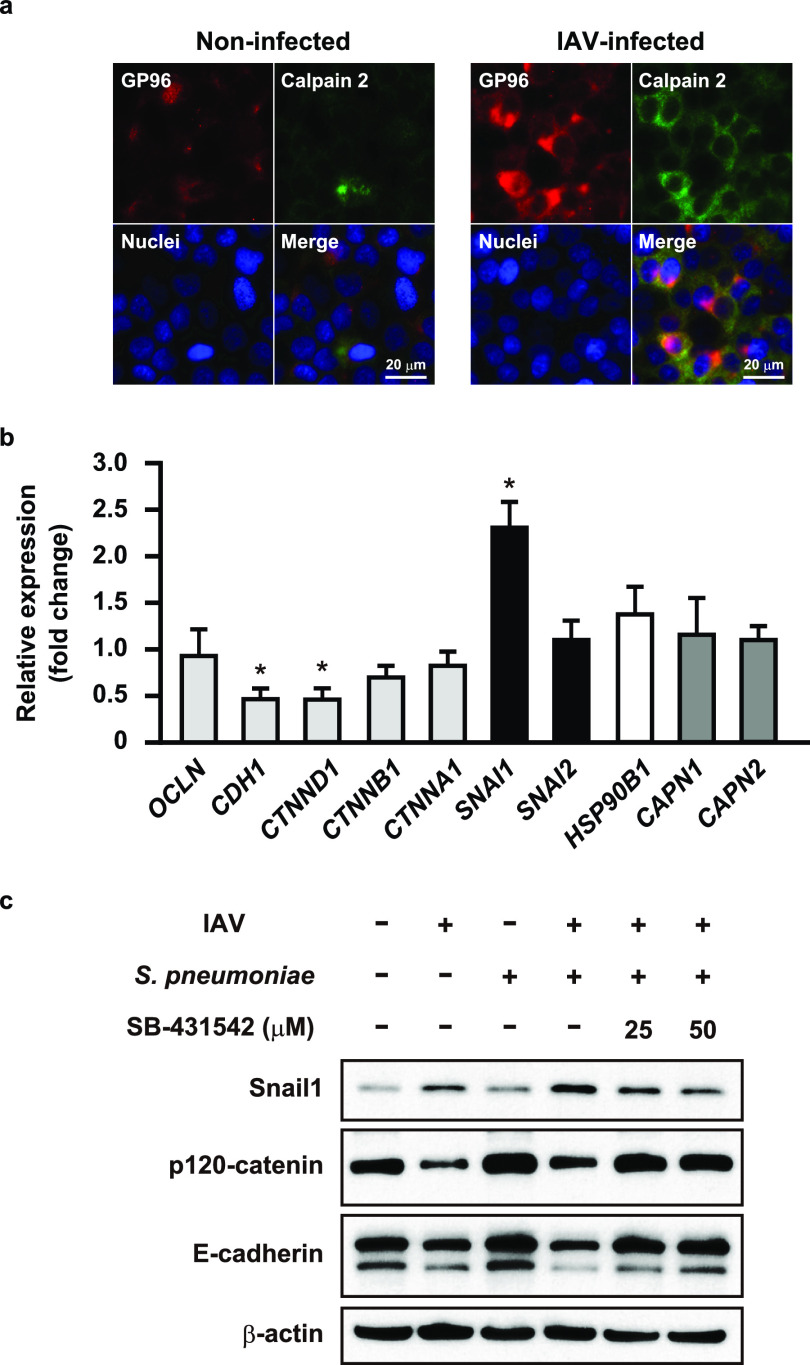
Calpain and Snail1 related to destruction of alveolar epithelial barrier following IAV infection. (a) A549 cells were infected with IAV for 1 h. After transferring to fresh medium, incubation was continued for 36 h. GP96 was labeled with anti-GP96 and Alexa Fluor 594-conjugated antibodies, while calpains were labeled with anti-calpain 2 and Alexa Fluor 488-conjugated antibodies. DAPI was used to stain DNA in the nucleus. (b) Transcriptional levels of genes encoding junctional proteins and regulators in A549 cells infected with IAV were analyzed using real-time RT-PCR. The *gapdh* transcript served as an internal control. *OCLN*, occludin; *CDH1*, E-cadherin; *CTNND1*, p120-catenin; *CTNNB1*, β-catenin; *CTNNA1*, αE-catenin; *SNAI1*, Snail1; *SNAI2*, slug; *HSP90B1*, GP96; *CAPN1*, calpain 1; *CAPN2*, calpain 2. Data from three independent tests are presented, with values shown as the means ± SDs for expression ratios. Transcriptional levels of tested genes are presented as relative expression levels normalized to that of noninfected cells. *, *P* < 0.01 compared to noninfected group. (c) A549 cells were infected with IAV for 1 h and then incubated with fresh medium in the presence or absence of SB-431542. Following the washing steps, cells were infected with an S. pneumoniae strain for 7 h. Expressions of E-cadherin, p120-catenin, and Snail1 were detected in whole-cell lysates using western blot analysis. β-Actin served as a loading control. All data shown are representative of at least three separate experiments.

Calcium (Ca^2+^) signaling has been implicated in various stages of host-pathogen interactions during viral and bacterial infections. Indeed, previous studies have shown that IAV infection induces Ca^2+^ influx, and then elevated intracellular Ca^2+^ promotes endocytic uptake of the virus and host inflammatory response (27, 28). Also, Ca^2+^ influx activates calpains, Ca^2+^-dependent host cysteine proteases, which then target junctional proteins, such as occludin and E-cadherin (29). Indeed, we previously found that calpain is recruited to the plasma membrane along with E-cadherin following S. pyogenes infection (30). Since calpains have been shown to change their distribution in an active state (31), cellular localization of GP96 and calpains in IAV-infected alveolar epithelial cells was assessed using immunofluorescence staining (Fig. 4a). As opposed to GP96, calpain 2 was not only exposed on the apical surface of epithelial cells but also found to be concentrated around the plasma membrane region after IAV infection, similar to that observed in S. pyogenes-infected cells. Our observations suggest that IAV infection recruits calpains to the plasma membrane of paracellular junctions, which in turn promotes destabilization of the alveolar epithelial barrier through degradation of junctional proteins. We then evaluated the effects of IAV infection on expressions of GP96 and calpains as well as junctional proteins associated with epithelial barrier function by using quantitative reverse transcription-PCR (RT-PCR) analysis. Distinct downregulation of E-cadherin and p120-catenin was detected in IAV-infected cells, whereas that infection had no effect on expression of GP96 or calpains at the transcriptional level (Fig. 4b). These results imply that IAV infection does not induce increased expression levels of GP96 and calpains but rather results in their ectopic localization in epithelial cells.

Interestingly, IAV infection induced a drastic upregulation of the host transcriptional factor Snail1, a global repressor of genes encoding junctional proteins (32, 33). Transforming growth factor β (TGF-β) has been shown to downregulate the expression of E-cadherin via the snail signaling pathway, known to be fundamental for development of epithelial-to-mesenchymal transition (EMT) (34). To investigate the association of Snail1 expression with destabilization of junctional proteins following infection, alveolar epithelial cells were infected with IAV in the presence or absence of a TGF-β inhibitor and then subsequently infected with S. pneumoniae (Fig. 4c). The protein level of Snail1 was elevated during both IAV infection and superinfection, whereas inhibition of the TGF-β pathway by SB-431542 in the coinfected cells resulted in remarkably reduced Snail1 levels in a dose-dependent manner. Along with increased levels of Snail1, reduced expression levels of E-cadherin and p120-catenin were observed in both IAV-infected and coinfected cells, while those were completely restored in the presence of the TGF-β inhibitor. Together, these data suggest that IAV infection induces Snail1-dependent dysfunction of the alveolar epithelial barrier via the TGF-β signaling pathway, thus providing a route for secondary pneumococcal dissemination.

### GP96 is involved in exacerbation of bacterial pneumonia following influenza virus infection.

**FIG 5:**
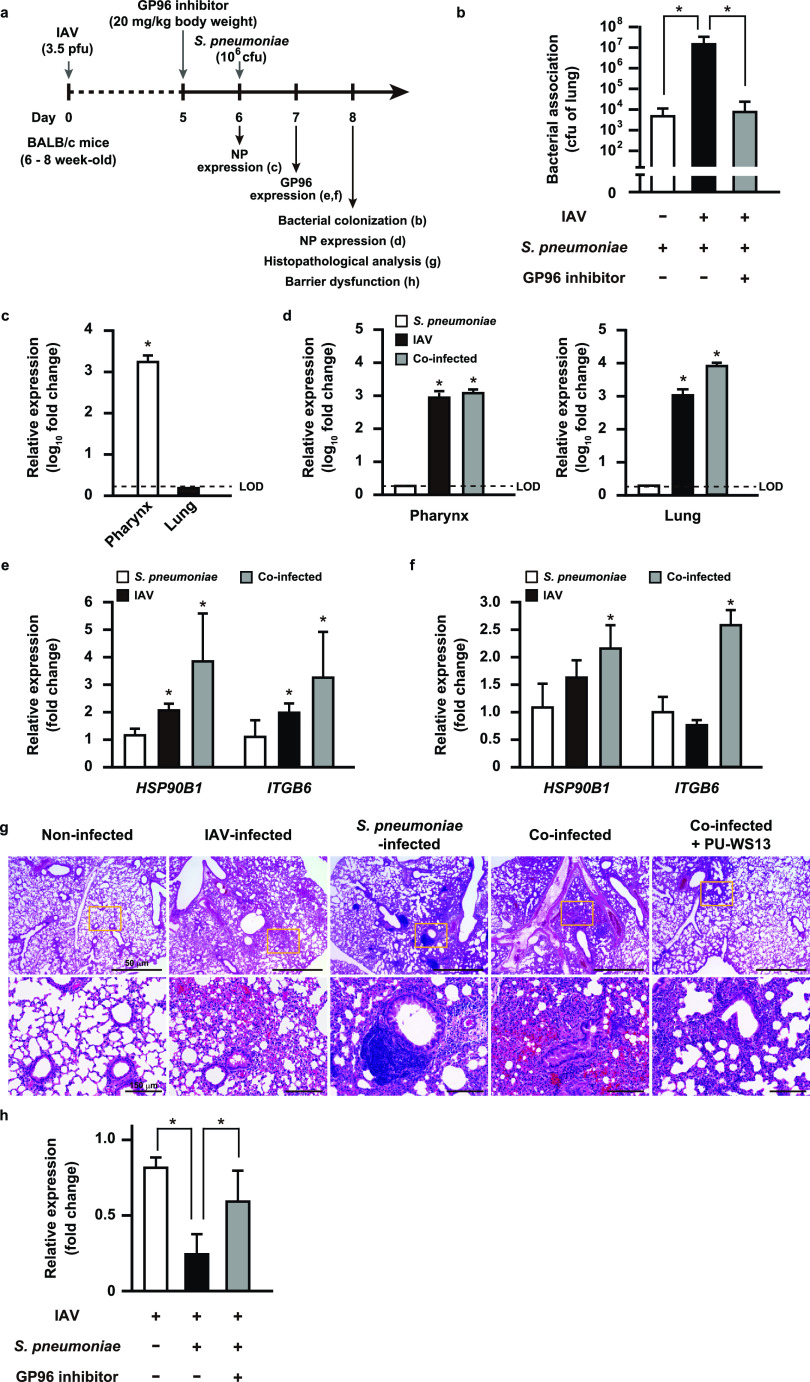
GP96 is a crucial factor for exacerbation of bacterial pneumonia following IAV infection. (a) Schematic showing experimental design. Mice were intranasally infected with IAV (day 0) followed by S. pneumoniae on day 6. In some experiments, PU-WS13, a GP96 inhibitor, was intratracheally administered. (b) Effect of PU-WS13 treatment on bacterial burden in lungs. Values shown represent the means ± SDs from quintuplet samples and are representative of at least three independent experiments. *, *P* < 0.01. Transcriptional levels of the *NP* gene encoding viral nucleoprotein in pharyngeal and lung tissues at 6 (c) or 8 (d) days after infection were analyzed using real-time RT-PCR. LOD, limit of detection. Transcriptional levels of *HSP90B1* and *ITGB6* genes encoding GP96 and integrin β_6_, respectively, in pharyngeal (e) and lung tissues (f) infected with IAV and S. pneumoniae were analyzed using real-time RT-PCR. The *gapdh* transcript served as an internal control. Values from three independent tests are presented as the means ± SDs for expression ratios (c to f). *, *P* < 0.01 compared to the noninfected group. (g) Lung tissues obtained from mice infected under various conditions were subjected to hematoxylin and eosin staining. Boxed areas are magnified and shown at the bottom. (h) Transcriptional levels of the E-cadherin gene in lung tissues infected under various conditions were analyzed by real-time RT-PCR. The *gapdh* transcript served as an internal control. Values from three independent tests are shown as the means ± SDs for expression ratios. Transcriptional levels are presented as relative expression levels normalized to that of noninfected tissues (c, d, e, f, and h). *, *P* < 0.01.

To further clarify the role of IAV-induced GP96 in secondary pneumococcal pneumonia *in vivo*, mice were intranasally infected with a nonlethal dose of IAV (day 0), which was followed by intratracheal administration of the vehicle or GP96 inhibitor PU-WS13 on day 5; then, intranasal challenge with S. pneumoniae was performed on day 6 (Fig. 5a). At 2 days after bacterial infection, coinfected mice showed a significantly greater bacterial burden in the lungs than those infected with S. pneumoniae only (Fig. 5b). Indeed, efficient viral replication in the pharyngeal tissues was found to be restricted at 6 days after IAV infection (Fig. 5c), whereas superinfection facilitated viral dissemination to lung tissues (Fig. 5d). Notably, PU-WS13 treatment resulted in a remarkable reduction in bacterial colonization in the lungs of the coinfected mice (Fig. 5b), indicating that mediation of GP96 induction by IAV infection has a key role in the pathogenesis of secondary bacterial pneumonia *in vivo*. Among integrin subsets, integrin α_V_β_6_ is an epithelium-restricted molecule expressed at low levels in the lungs of healthy individuals and then becomes rapidly upregulated in response to inflammation and injury. Meliopoulos et al. reported that integrin β_6_ is an important factor associated with the severity of influenza diseases (35). To further elucidate the mechanism by which IAV infection leads to increased susceptibility to secondary bacterial pneumonia, we examined the expression levels of integrin β_6_ and GP96 in pharyngeal and lung tissues at 1 day after bacterial administration under each infection condition (Fig. 5e and f). Quantitative RT-PCR analysis showed that IAV infection resulted in an approximately 2-fold increase in expression levels of GP96 and integrin β_6_ in pharyngeal tissues (Fig. 5e) but not in lung tissues (Fig. 5f). Of note, high expression levels of GP96 and integrin β_6_ were detected in both pharyngeal and lung tissues after superinfection. These results suggest that pneumococcal colonization in the IAV-infected upper respiratory tract triggers GP96 expression in lung tissues, which in turn allows bacterial dissemination to the lower respiratory tract. Histopathological analysis was also performed using lungs obtained from mice at 2 days after pneumococcal infection (Fig. 5g). In lung tissues infected with IAV alone, moderate levels of inflammatory cell infiltration in peribronchiolar and interalveolar spaces as well as microvascular hemorrhage were observed. Mice infected with S. pneumoniae alone also showed prominent perivascular and peribronchiolar lymphocytic cuffing. In contrast, mice with coinfection demonstrated dense leukocytic infiltration in interstitial and alveolar spaces along with hemorrhaging, vascular leakage, and edema formation, suggesting vascular damage and increased epithelial-endothelial permeability. Notably, PU-WS13 treatment resulted in improvement of lung pathology in coinfected mice. To investigate lung tissue integrity in superinfected mice, we examined the expression of E-cadherin in lung tissues obtained from IAV-infected as well as coinfected mice. Those with coinfection exhibited a marked decrease in E-cadherin expression compared to that in mice infected with IAV alone and was partially restored by PU-WS13 treatment (Fig. 5h). Taken together, our results indicate that GP96 is a factor for exacerbation of secondary bacterial pneumonia following influenza as well as a promising novel target for therapeutic intervention.

## DISCUSSION

Secondary bacterial infections following a primary influenza virus infection are frequent complications and result in the majority of related deaths during seasonal and pandemic influenza outbreaks. S. pneumoniae is the most commonly identified pathogen in secondary bacterial pneumonia cases. Although antibiotics remain the mainstay of therapy for affected patients, the increasing prevalence of multidrug-resistant S. pneumoniae is a serious public health concern worldwide. Thus, the development of host-directed therapeutics is receiving focus as an alternative approach to treating secondary bacterial pneumonia following influenza. The present findings showed that GP96 functions as an exacerbation factor for secondary bacterial infections following influenza; thus, we propose GP96 as a potential therapeutic target for novel countermeasures used to treat bacterial pneumonia.

GP96, an endoplasmic reticulum (ER)-resident HSP90 paralogue, has been reported to be exposed on the surfaces of various types of cells by multiple types of microbial infection (16). The present is the first study to show that IAV infection triggers surface distribution of GP96 on human airway epithelial cells, where it is then hijacked as a host receptor for secondary infection by S. pneumoniae. The interaction between extracellular GP96 and bacterial surface ligands has been shown to activate host signaling cascades that facilitate bacterial adherence and internalization. Indeed, pathogenic Escherichia coli targets Ecgp96, a homologue of GP96 expressed on human brain microvascular endothelial cells, and induces rearrangement of actin microfilaments and disassembly of endothelial junctions through signaling-mediated Ca^2+^ influx and nitric oxide production, resulting in an acceleration of bacterial invasion (36, 37). Bacterial pore-forming toxins such as pneumolysin produced by S. pneumoniae also induce similar cellular events in host cells (38). Although the present results demonstrated that IAV infection induces Ca^2+^-dependent calpain activation, which then evokes destabilization of paracellular junctions without bacterial infection, S. pneumoniae may utilize not only extracellular GP96-mediated signaling but also pneumolysin-induced cell damage for invasion into deeper tissues. Despite increased understanding regarding the roles of GP96 in the pathogenesis of infections, the molecular mechanisms underlying surface distribution remain unidentified. Recently, plasma membrane damage mediated by bacterial pore-forming toxins was shown to be associated with the redistribution of GP96 via nonmuscle myosin II activity and Ca^2+^ influx during L. monocytogenes infection (39). Although the mechanism governing the surface distribution of GP96 following IAV infection remains largely unknown, it is likely that calcium homeostasis and Ca^2+^-dependent effectors have key roles in IAV-infected airway epithelial cells.

Bacterial colonization in the upper respiratory tract is considered to be a prerequisite for invasive infection, which results in bacterial invasion into other tissues or dissemination to the lower respiratory tract. In addition to a drastic increase in receptor availability accompanied by influenza virus infection, S. pneumoniae also possesses a variety of adhesins that augment bacterial adherence to these newly exposed receptors (40). Here, we identified AliA and AliB, oligopeptide-binding proteins, as bacterial adhesins for GP96 on the surface of alveolar epithelial cells following IAV infection. The Ami-AliA/AliB permease complex is composed of three oligopeptide-binding proteins, AmiA, AliA, and AliB, two transmembrane proteins, AmiC and AmiD, and two ATPases, AmiE and AmiF (26). AmiA, AliA, and AliB bind to and utilize peptides located in ribosomal proteins of commensal bacterial species in the nostrils and nasopharynx for establishment of pneumococcal colonization in host niches (41). These oligopeptide-binding proteins share a homology with a >60% amino acid identity; thus, they have overlapping specificity to some substrates (26, 41). Based on that previous report as well as the present findings, it is considered likely that both AliA and AliB are responsible for GP96 binding. On the other hand, a compensation of deficiency in function with either *aliA* or *aliB* deletion was not observed in regard to the pneumococcal association with IAV-infected cells. One possibility for this discrepancy is that deletion of either *aliA* or *aliB* induced a conformational change that restricted access by GP96 to the other Ali protein. Nevertheless, the rate of association of the *aliA* or *aliB* deletion mutant with IAV-infected cells was decreased compared to that of the wild type; thus, the Ami-AliA/AliB permease complex is considered to contribute to pneumococcal adherence thorough GP96. In addition, these oligopeptide-binding proteins are conserved among bacterial pathogens most frequently associated with influenza, including S. pneumoniae, S. pyogenes, S. aureus, and H. influenzae. GP96 serves as the host cellular receptor for various bacterial adhesins, such as pathogenic E. coli OmpA (36, 37), L. monocytogenes Vip (17), S. aureus Bap (42), and Clostridium difficile enterotoxin A (43), though those proteins share no homology with Ali proteins. Since GP96 might nonspecifically interact with multiple bacterial molecules on account of its chaperone structure, bacterial pathogens likely utilize ectopically exposed GP96 for the establishment of bacterial colonization.

TGF-β, a multifunctional cytokine, is secreted in an inactive or latent form and then subsequently activated through various mechanisms. During IAV infection, viral neuraminidase was shown to activate TGF-β, which promoted upregulation of host adhesion molecules, including fibronectin and integrins (44). TGF-β is also a positive regulator of the integrin signaling pathway that promotes cytoskeletal rearrangement and bacterial internalization (45). Indeed, we previously reported that S. pyogenes possesses various Fn-binding molecules and utilizes Fn integrin interactions to adhere to and invade IAV-infected cells (46). On the other hand, only a few types of Fn-binding proteins are exposed on the surface of S. pneumoniae. Based on our observations that the bacterial association was only partially reduced by treatment with an anti-integrin α_V_ antibody, integrins may function as an ancillary but not major receptor in secondary pneumococcal infection. In addition to TGF-β signaling, the present findings showed that IAV infection induces upregulation and display of integrins on the surface of alveolar epithelial cells via chaperone activity of GP96. Furthermore, GP96 serves as an essential chaperone for the cell-surface protein glycoprotein A repetitions predominant (GARP), a docking receptor for latent membrane-associated TGF-β (47), indicating GP96 as a crucial factor for TGF-β signaling. Notably, S. pneumoniae also expresses neuraminidases on bacterial cell walls. NanA, a primary pneumococcal neuraminidase, is a sialidase that catalyzes cleavage of terminal sialic acids from latency-associated peptide and also activates TGF-β signaling (48). Another study showed that activation of TGF-β signaling proceeds through phosphorylation of SMAD proteins, which is associated with Snail1-mediated downregulation of tight junction proteins of epithelial and endothelial cells (49). The present results provide evidence that a preceding influenza virus infection induces Snail1-dependent dysfunction of the airway epithelial barrier through TGF-β signaling, thus preparing a route for secondary pneumococcal translocation into deeper tissues via paracellular junctions. Therefore, the synergistic effects of the GP96 chaperone function and viral-pneumococcal neuraminidase activities likely prime potent TGF-β signaling, which leads to increased bacterial loading in the lungs and pulmonary barrier dysfunction.

Besides being exploited as a host receptor for a variety of pathogens, GP96 modulates the host immune response to counteract an infection. GP96 functions as a master chaperone for cellular localization and function of TLRs. During an influenza virus infection, TLRs act as key transducers of type I interferons (IFNs) by recognition of viral nucleic acid (7). It is known that type I IFN signaling through the IFN-α/β receptor evokes expression of proinflammatory genes to inhibit viral replication and augment various aspects of adaptive immunity, while several lines of evidence also indicate that neutrophil function is impaired following influenza virus infection. Viral infection-primed expression of type I IFNs is sufficient to interfere with production of specific chemokines, such as CXCL1 and CXCL2, resulting in impairment of the neutrophil response during secondary S. pneumoniae infection (50). The present findings showed that pneumococcal colonization in the upper respiratory tract triggered upregulation of GP96 through an unidentified mechanism in murine pharyngeal and lung tissues infected with IAV. Accordingly, given the importance of GP96 in TLR signaling (20), IAV infection-induced GP96 might impair immune responses against S. pneumoniae by excess production of type I IFNs. On the other hand, GP96 has been shown to specifically bind to and activate neutrophils and monocytes (51). Nevertheless, we speculate that binding of pneumococcal Ali proteins to GP96 interferes with direct interactions of GP96 with neutrophils as well as monocytes in cases of viral-bacterial dual infection. Although direct evidence showing that GP96 is related to impairment in macrophages and neutrophil responses following IAV infection remains lacking, it may function as a potent receptor for bacterial colonization as well as an immune regulator in dysfunction of innate immune defenses against bacterial infection.

Taken together, the present results indicate that GP96 functions as a multifunctional exacerbation factor to promote pneumococcal colonization, dysfunction of lung tissue and barrier responses, and probably dysregulation of immune responses as well, during secondary bacterial pneumonia following an influenza virus infection. Because of the complexity of the pathogenesis, a balanced combination of antimicrobial agents and immunomodulators could be more effective for prospective therapeutics. We believe that GP96 is a potential target for the development of promising therapeutic strategies, including combination therapies as alternatives to conventional antibiotics and antiviral agents administered for broad-spectrum prevention, as well as management of secondary bacterial infections following influenza.

## MATERIALS AND METHODS

### Bacterial and viral strains and culture conditions.

Streptococcus pneumoniae D39 (serotype 2 clinical isolate) and isogenic mutant strains were cultured in Todd-Hewitt broth (Becton, Dickinson and Company [BD]) supplemented with 0.2% yeast extract (BD) (THY medium) at 37°C in an ambient atmosphere. For selection and maintenance of mutant strains, spectinomycin (Sigma-Aldrich) at 100 μg/ml was added to the medium. Escherichia coli strains BL21-gold(DE3) (Agilent Technologies) and XL10-gold (Stratagene) served as hosts for derivatives of plasmids pGEX-6P-1 (Cytiva) and pQE30 (Qiagen), respectively. All E. coli strains were cultured in Luria-Bertani (Nacalai Tesque) (LB) medium at 37°C with agitation. For selection and maintenance of E. coli mutant strains, ampicillin (100 μg/ml) was added to the medium. Influenza A virus strains, A/FM/1/47 (H1N1) and A/Panama/2007/99 (H3N2), were grown in Madin-Darby canine kidney (MDCK) cells.

### Cell cultures and construction of GP96 knockout cells.

The human alveolar carcinoma cell line A549 (Riken Cell Bank) derived from type II pneumocytes and MDCK cells were maintained in DMEM supplemented with 10% FBS at 37°C under a 5% CO_2_ atmosphere.

A CRISPR-Cas9 GP96 knockout plasmid was created by cloning single guide RNA (sgRNA) GP96 oligonucleotides into a pSpCas9(BB)-2A-Puro (PX459) plasmid, a gift from Feng Zhang (Addgene plasmid 62988) (52). The constructed plasmid was then transfected into A549 cells with Lipofectamine 3000 reagent (Thermo Scientific), according to the manufacturer’s instructions. At 24 h after transfection, 1.5 μg/ml puromycin was added and the cells were further cultured for 2 days. Next, a limiting dilution of the survived cells was conducted and single colonies were expanded, with GP96 expression examined by western blotting using anti-Grp94 rabbit antibody (Ab) (Cell Signaling). PCR products were amplified with purified genomic DNA and the primers gp96checkF and gp96checkR and then subjected to Sanger sequencing for confirmation of mutations. Cells with no mutation of the GP96 gene, transfected with the plasmid, and cultured in the same manner as the GP96 knockout cells during selection were used as control cells. All primers used are listed in Table S1 in the supplemental material.

10.1128/mBio.03269-20.1 TABLE S1 Oligonucleotides used in this study. Download Table S1, DOCX file, 0.1 MB. Copyright © 2021 Sumitomo et al. 2021 Sumitomo et al. https://creativecommons.org/licenses/by/4.0/ This content is distributed under the terms of the Creative Commons Attribution 4.0 International license.

### Preparation of S. pneumoniae mutant strains and recombinant proteins.

Inactivation of the *aliA* and *aliB* genes was achieved by transformation of strain D39 with a linear DNA fragment containing a spectinomycin resistance gene (*aad9*) flanked by the upstream and downstream sequences of the *aliA* and *aliB* genes, as previously reported (53).

For construction of recombinant GP96, cDNA of A549 cells was prepared using TRIzol and a PureLink RNA minikit (Thermo Scientific). cDNA fragments encoding full-length GP96 were amplified using specific primers (Table S1). The fragments were cloned into a pGEX-6P-1 vector via the BamHI and SalI sites and then transformed into E. coli BL21(DE3).

Recombinant AliA and AliB proteins were hyperexpressed in E. coli XL10-Gold using a pQE30 vector. N-terminal His-tagged proteins were purified using a QIAexpress protein purification system (Qiagen), as previously described (54).

### Adherence assay.

A549 cells were cultured in 24-well plates at a density of 2 × 10^5^ cells per well and infected with 10^6^ PFU of IAV in serum-free DMEM supplemented with 0.1% bovine serum albumin (BSA) (Sigma-Aldrich), MEM vitamin solution (Thermo Scientific), and 0.01% DEAE dextran (Gibco) for 1 h at 34°C. Following the washing steps, the cells were incubated for 36 h in the presence or absence of PU-WS13 (Merck Millipore). S. pneumoniae strains were grown to the exponential phase (optical density at 600 nm [OD_600_] of 0.7) and then washed with and resuspended in phosphate-buffered saline (PBS). IAV-infected cells were exposed to 10^7^ CFU of pneumococci in DMEM supplemented with 10% FBS for 2 h at 37°C. For quantification of bacterial adherence, infected cells were washed with PBS and lysed with distilled water. Serial dilutions of the lysates were plated on THY agar plates to determine CFU.

In some experiments, antibodies targeting Grp94 (rat monoclonal antibody [MAb]; Enzo) and integrin α_V_ (goat polyclonal antibody [PAb]; R&D), rat IgG2A isotype control (rat MAb; R&D), normal goat IgG control (goat PAb; Enzo), RGD peptide (Gly-Arg-Gly-Asp-Asn-Pro; Enzo), and RGD control peptide (Gly-Arg-Ala-Asp-Ser-Pro; Enzo) were added to A549 cells 1 h before infection with S. pneumoniae.

### Immunoprecipitation assay.

Cell surface proteins were biotinylated using an EZ-Link sulfo-*N*-hydroxysuccinimide [NHS]-biotin reagent (Thermo Scientific) according to the manufacturer’s protocol. Briefly, noninfected and IAV-infected A549 cells were washed with PBS and then incubated with 1 mM sulfo-NHS-biotin for 30 min at room temperature. After quenching the biotin reagent with 100 mM glycine in PBS, the cells were lysed using radioimmunoprecipitation buffer containing cOmplete protease inhibitor (Roche Life Science) for 20 min at 4°C. Cell surface proteins were precipitated with Dynabeads M-280 streptavidin (Thermo Scientific), separated using SDS-PAGE, and then visualized by silver staining. Bands of interest were analyzed by liquid chromatography-tandem mass spectrometry using a Q-Exactive mass spectrometer (Thermo Scientific) equipped with an UltiMate 3000 nanoscale liquid chromatography (nanoLC) system (Thermo Scientific). Raw data were processed using Mascot Distiller v2.5 (Matrix Science). Peptide and protein identification was performed with Mascot, v.2.5 (Matrix Science), using the UniProt database with a precursor mass tolerance of 10 ppm, a fragment ion mass tolerance of 0.01 Da, and strict trypsin specificity that allowed one missed cleavage. To determine cellular localization of integrin α_V_, immunoprecipitation using an antibody against integrin α_V_ (rabbit PAb; Cell signaling) and Dynabeads protein A (Thermo Scientific) was performed, and then detection was performed by western blotting with a specific antibody against integrin α_V_ (goat PAb; R&D) and a horseradish peroxidase (HRP)-conjugated antibody against goat IgG (R&D) or HRP-conjugated streptavidin (Thermo Scientific). Immunoreactive bands were detected using Pierce western blotting substrate (Thermo Scientific).

For identification of bacterial factors associated with GP96, cell wall fractions of S. pneumoniae were prepared as previously described (55). The fractions were incubated with 10 μg of recombinant GP96 for 6 h at 4°C. Proteins bound to GP96 were immunoprecipitated with an antibody against Grp94 (rat MAb; Enzo) and Dynabeads protein G (Thermo Scientific) and then examined using mass spectrometry analysis, as described above.

### Surface plasmon resonance analysis.

Association and dissociation reactions of GP96 to pneumococcal Ali proteins were analyzed using a BIAcore optical biosensor (BIAcore X-100 system; GE Healthcare Life Sciences), as previously described (48). Briefly, recombinant GP96 (20 μg ml^−1^ in 10 mM sodium acetate, pH 4) was covalently immobilized on a CM5 sensor chip using an Amine coupling kit (GE Healthcare Life Sciences). Binding analyses were performed in HBS-P buffer (0.01 M HEPES [pH 7.4], 0.15 M NaCl, 0.005% surfactant P20; GE Healthcare Life Sciences) at 37°C with a flow rate of 30 μl/min. AliA and AliB were separately used as an analyte at concentrations of 31.3, 62.5, 125, 250, and 500 nM. Parameters of binding kinetics were analyzed using raw data from the BIAcore sensorgram suitable for analysis with the kinetic models included in the BIA evaluation software package, v. 3.0.2 (GE Healthcare Life Sciences). Data were fitted using a 1:1 Langmuir binding model.

### ELISA.

GP96 binding to AliA and AliB proteins was assessed by enzyme-linked immunosorbent assay (ELISA), as previously described (50). Microtiter plates (96-well; Sumitomo Bakelite) were separately coated with AliA, AliB, and PhtD protein (250 ng) in coating buffer (0.1 M Na_2_CO_3_, 0.1 M NaHCO_3_, pH 9.6) at 4°C overnight. The plates were blocked with 10% Block Ace solution (Megmilk Snow Brand) at 4°C overnight and then washed with PBS containing 0.2% Tween 20 (PBST). GP96 was diluted with binding buffer (50 mM HEPES [pH 7.4], 150 mM NaCl, 2 mM CaCl_2_, 50 μg/ml BSA) and incubated with immobilized bacterial surface proteins for 90 min at 37°C. After washing with PBST, the plates were incubated with an antibody against GP96 (sheep PAb; R&D) for 2 h at room temperature. Subsequently, an HRP-conjugated antibody against sheep IgG (R&D) was added to the plate, and incubation was continued for 2 h at room temperature. Following a washing step, the peroxidase substrate tetramethylbenzidine (Moss) was added to the plate. The reaction was stopped by the addition of 0.5 N HCl, and absorbance at 450 nm was measured using a Multiskan FC microplate photometer (Thermo Scientific).

### Immunofluorescence microscopy.

A549 cells were seeded at 2 × 10^5^ onto coverslides (13-mm diameter; Matsunami) pretreated with coating buffer containing 0.1% collagen (type I from rat tail; Sigma-Aldrich) and 0.1% gelatin (from bovine bone; Wako). The cells were then infected with IAV followed by S. pneumoniae, as described above, and fixed with 4% paraformaldehyde-PBS. Following blocking with 5% bovine serum albumin-PBS, the cells were reacted with a primary antibody targeting Grp94 (rat MAb, Enzo), Grp94 (rabbit PAb; Thermo Scientific), integrin α_V_ (mouse MAb; R&D), calpain 2 (rabbit PAb; Cell Signaling), or serotype 2 capsule (rabbit PAb; Denka Seiken). After the washing steps, incubation was performed with Alexa Fluor 594-conjugated anti-rat IgG (Thermo Scientific), Alexa Fluor 594-conjugated anti-mouse IgG (Thermo Scientific), Alexa Fluor 488-conjugated anti-mouse IgG (Thermo Scientific), or Alexa Fluor 488-conjugated anti-rabbit IgG (Thermo Scientific). To observe the association of pneumococci with GP96, imaging was performed using a Zeiss LSM 510 confocal microscope system, v. 3.2 (Carl Zeiss) and analyzed with the LSM 510 software package. The signal intensity profile for each channel was evaluated using ImageJ 1.53e (NIH). For assessment of surface display of GP96, integrin α_V_, and calpain 2, coverslides were enclosed with ProLong Gold antifade reagent with 4′,6-diamidino-2-phenylindole (DAPI) (Thermo Scientific) and examined using a Carl Zeiss Axioplan 2 fluorescence microscope system.

### Analysis of destabilization of epithelial junctions.

Whole-cell lysates from coinfected epithelial cells were prepared as previously described (55). Briefly, A549 cells were infected with IAV in the presence or absence of SB-431542, a TGF-β inhibitor. At 7 h after infection, the cells were lysed with Laemmli gel loading buffer containing 6% 2-mercaptoethanol. Cleavage of junctional proteins was detected by western blotting using specific antibodies against E-cadherin (mouse MAb; Thermo Scientific), p120-catenin (rabbit PAb; Cell Signaling), Snail1 (mouse MAb; Cell Signaling), and β-actin (rabbit PAb; Cell Signaling). Horseradish peroxidase (HRP)-conjugated antibodies against mouse or rabbit IgG (Cell Signaling) were used as the secondary antibodies.

### Mouse experiments.

Female BALB/c mice at 6 to 8 weeks old (CLEA Japan, Inc.) were intranasally infected with 3.5 PFU of IAV A/FM/1/47 (H1N1) in 40 μl PBS (day 0). The S. pneumoniae D39 strain was grown to the mid-exponential phase (OD_600_ of 0.4) and then washed with and resuspended in PBS. Bacteria were introduced into the mice by intranasal administration of 1 × 10^6^ CFU in 40 μl PBS on day six. In some experiments, mice were intratracheally treated with either the vehicle or PU-WS13 (20 mg/kg mouse body weight) on day five.

For quantification of bacterial colonization in the lung, mice were euthanized 2 days after S. pneumoniae infection, and then lung tissues were immediately collected. Lung homogenates were serially diluted and plated on THY agar plates containing 5% sheep blood. For histopathologic examinations, lung tissue samples were obtained and fixed with formalin, embedded in paraffin, sectioned, and subjected to hematoxylin and eosin (HE) staining. Stained tissue sections were observed using an EVOS M5000 cell imaging system (Thermo Scientific). For assessment of gene expression, pharyngeal and lung tissues were harvested at various time points.

### Real-time RT-PCR assay.

Total RNA was isolated from A549 cells using a CellAmp Direct RNA prep kit (TaKaRa) as well as from murine pharyngeal and lung tissues using an RNeasy fibrous tissue minikit (Qiagen). Synthesis of cDNA from total RNA was performed with a PrimeScript RT reagent kit (TaKaRa). The possibility of DNA contamination was excluded by PCR analysis of non-RT samples. Primer sets for selected genes were designed using Primer Express software, version. 3.0 (Applied Biosystems). All primers used are listed in Table S1. RT-PCR amplifications were performed using the SYBR green method with an ABI StepOne real-time PCR system, v. 2.2 (Applied Biosystems). Relative expression amounts were calculated with the comparative threshold cycle (ΔΔ*C_T_*) method. The level of *gapdh* expression was used as an internal control.

### Statistical analysis.

All statistical analyses were performed using GraphPad Prism, v. 7.03 (GraphPad Software). Differences were determined with Mann-Whitney’s *U* test when comparing two groups or one-way analysis of variance (ANOVA) followed by Tukey’s multiple-comparison test when comparing multiple groups. A confidence interval with a *P* value of <0.01 was considered to be significant.

### Ethics statement.

All mouse experiments were conducted using a protocol approved by the Animal Care and Use Committee of Osaka University Graduate School of Dentistry (authorization number 01-010-0) and the Animal Care and Use Committee of Kanazawa University (authorization numbers AP-143262 and AP-183936).
