# Supplementary material for: GP96 Drives Exacerbation of Secondary Bacterial Pneumonia following Influenza A Virus Infection
Source: mBio. 2021 Jun 1;12(3):e03269-20. doi: 10.1128/mBio.03269-20 (PMC8262878; doi:10.1128/mBio.03269-20)
Supplement: TABLE S1 [file mbio.03269-20-st001.docx]

**Sumitomo *et al.* Supplemental table 1**

Table S1. Oligonucleotides used in this study.

Primer Sequence (5’-3’) Purpose

gp96koF CACCGTCATTCTGTTAACTTCGGCT deletion of the *gp96* gene

gp96koR AAACAGCCGAAGTTAACAGAATGAC deletion of the *gp96* gene

gp96checkF ACTTTGCTTTTTAGTAGAGGAA confirmation of the *gp96* deletion

gp96checkR TTACTTTTGCAGTAAATTAAG confirmation of the *gp96* deletion

aliAkoF1 TTACGCTTTACCATCATGATCAGC deletion of the *aliA* gene

aliAkoR1 CTTTATTAATTTGTTCGTATGTATTCATCATTTTCTCCTTTAAAACTTTCTCTCC deletion of the *aliA* gene

aliAkoF2 GGAGAGAAAGTTTTAAAGGAGAAAATGATGAATACATACGAACAAATTAATAAAG deletion of the *aliA* gene

aliAkoR2 CTAAATCCTTTCTTATATTTTGCAACAGTTATAATTTTTTTAATCTGTTATTTAAATAG deletion of the *aliA* gene

aliAkoF3 CTATTTAAATAACAGATTAAAAAAATTATAACTGTTGCAAAATATAAGAAAGGATTTAG deletion of the *aliA* gene

aliAkoR3 ACGACTACCTCTATACCAAGTGC deletion of the *aliA* gene

aliAkockF GTTAGAACAACCTCTTCTTCATGC confirmation of the *aliA* deletion

aliAkockR ACTGTGTCAAAGAGATTGACATCG confirmation of the *aliA* deletion

aliBkoF1 CCGATAACCCCCATAGGTTAGC deletion of the *aliB* gene

aliBkoR2 CTATTTAAATAACAGATTAAAAAAATTATAAAATCTAATTGTAGATAAGTTTGTGTAAG deletion of the *aliB* gene

aliBkoF2 CTTACACAAACTTATCTACAATTAGATTTTATAATTTTTTTAATCTGTTATTTAAATAG deletion of the *aliB* gene

aliBkoR2 CAAATATTTAAAGCAGGAGGTTCTGGAAATGAATACATACGAACAAATTAATAAAG deletion of the *aliB* gene

aliBkoF3 CTTTATTAATTTGTTCGTATGTATTCATTTCCAGAACCTCCTGCTTTAAATATTTG deletion of the *aliB* gene

aliBkoR3 TCAGCCAATCCTAATAAAATCACG deletion of the *aliB* gene

aliBkockF CTAGAATAAACAGTTACAAAATTAGC confirmation of the *aliB* deletion

aliBkockR GCTCTTCCAGATTTTGGATCAGC confirmation of the *aliB* deletion

rGP96F CGGGATCCGACGATGAAGTTGATGTGGATGGTAC construction of rGP96

rGP96R GCGTCGACCTTTGCATCAGGGTCAATGTTC construction of rGP96

rAliAF CGCGAGCTCAAAGGTGAGAAGACATTCTCATAC construction of rAliA

rAliAR ACGCGTCGACTTTCACATGTTTTGCGAGATCTTC construction of rAliA

rAliBF CGCGAGCTCAATTCTAGCACTGCATCAAAAACC construction of rAliB

rAliBR ACGCGTCGACTTTGACATGTTTTGCCAATTCTTC construction of rAliB

OCLNF CACACAGGACGTGCCTTCAC real-time RT-PCR

OCLNR GAGTATGCCATGGGACTGTCAA real-time RT-PCR

CDH1F CCAGTGAACAACGATGGCATT real-time RT-PCR

CDH1R TGCTGCTTGGCCTCAAAAT real-time RT-PCR

CTNND1F TGCACTGCATGCCTTGACA real-time RT-PCR

CTNND1R GTTCCCGCTCCCAACCA real-time RT-PCR

CTNNB1F TGCCATTCCACGACTAGTTCAG real-time RT-PCR

CTNNB1R CGTACGGCGCTGGGTATC real-time RT-PCR

CTNNA1F GAATGTCTGCAAGCCAGTTAGAAG real-time RT-PCR

CTNNA1R TGCTAAAGCCAGTGCAGCAT real-time RT-PCR

SNAI1F TTCAACTGCAAATACTGCAACAAG real-time RT-PCR

SNAI1R GCGTGTGGCTTCGGATGT real-time RT-PCR

SNAI2F ACGCCCAGCTACCCAATG real-time RT-PCR

SNAI2R TCACTCGCCCCAAAGATGAG real-time RT-PCR

hHSP90B1F GCCCCCTAATCCCCTTCTC real-time RT-PCR

hHSP90B1R CCACTTTTTCCTGTGACCCATAA real-time RT-PCR

CAPN1F CACGACACCCTGATCTGAAGAC real-time RT-PCR

CAPN1R CCACCATGCTGCGACATG real-time RT-PCR

CAPN2F CCTGCTGGAGAAGGCATACG real-time RT-PCR

CAPN2R GGCACCCCCTGATAGTGCTT real-time RT-PCR

GAPDHF CGGACTTCCTCGGTGATACC real-time RT-PCR

GAPDHR CAATGCCGGCCTTAGCAT real-time RT-PCR

mHSP90B1F GTCAAAAGAAAACGTTCGAAATCA real-time RT-PCR

mHSP90B1R CCGCCGCAACATGTCTCT real-time RT-PCR

mITGB6F AAGGCCAAGTGGCAAACG real-time RT-PCR

mITGB6R CGTTCTTAAAAGTGCTGGTGGAA real-time RT-PCR

mCDH1F ACCCCCTTACGACTCTCTGTTG real-time RT-PCR

mCDH1R CAGGCTAGCGGCTTCAGAAC real-time RT-PCR

mGAPDHF CATGGCCTTCCGTGTTCCTA real-time RT-PCR

mGAPDHR GCGGCACGTCAGATCCA real-time RT-PCR

IAVNPF CAGCCTAATCAGACCAAATG real-time RT-PCR

IAVNPR TACCTGCTTCTCAGTTCAAG real-time RT-PCR
